# SNP array profiling of mouse cell lines identifies their strains of origin and reveals cross-contamination and widespread aneuploidy

**DOI:** 10.1186/1471-2164-15-847

**Published:** 2014-10-03

**Authors:** John P Didion, Ryan J Buus, Zohreh Naghashfar, David W Threadgill, Herbert C Morse, Fernando Pardo-Manuel de Villena

**Affiliations:** Department of Genetics, University of North Carolina at Chapel Hill, CB 7295, Chapel Hill, NC 27599-7264 USA; Lineberger Comprehensive Cancer Center, University of North Carolina at Chapel Hill, CB 7295, Chapel Hill, NC 27599-7264 USA; Carolina Center for Genome Science, University of North Carolina at Chapel Hill, CB 7295, Chapel Hill, NC 27599-7264 USA; Laboratory of Immunogenetics, National Institute of Allergy and Infectious Diseases, National Institutes of Health, Twinbrook I, Room 1421, 5640 Fishers Lane, Rockville, MD 20852 USA; Department of Veterinary Pathobiology, College of Veterinary Medicine and Biomedical Sciences, Texas A&M University, College Station, TX 77843 USA; Department of Molecular and Cellular Medicine, College of Medicine, Texas A&M University, College Station, TX 77843 USA

## Abstract

**Background:**

The crisis of Misidentified and contaminated cell lines have plagued the biological research community for decades. Some repositories and journals have heeded calls for mandatory authentication of human cell lines, yet misidentification of mouse cell lines has received little publicity despite their importance in sponsored research. Short tandem repeat (STR) profiling is the standard authentication method, but it may fail to distinguish cell lines derived from the same inbred strain of mice. Additionally, STR profiling does not reveal karyotypic changes that occur in some high-passage lines and may have functional consequences. Single nucleotide polymorphism (SNP) profiling has been suggested as a more accurate and versatile alternative to STR profiling; however, a high-throughput method for SNP-based authentication of mouse cell lines has not been described.

**Results:**

We have developed computational methods (Cell Line Authentication by SNP Profiling, CLASP) for cell line authentication and copy number analysis based on a cost-efficient SNP array, and we provide a reference database of commonly used mouse strains and cell lines. We show that CLASP readily discriminates among cell lines of diverse taxonomic origins, including multiple cell lines derived from a single inbred strain, intercross or wild caught mouse. CLASP is also capable of detecting contaminants present at concentrations as low as 5%. Of the 99 cell lines we tested, 15 exhibited substantial divergence from the reported genetic background. In all cases, we were able to distinguish whether the authentication failure was due to misidentification (one cell line, Ba/F3), the presence of multiple strain backgrounds (five cell lines), contamination by other cells and/or the presence of aneuploid chromosomes (nine cell lines).

**Conclusions:**

Misidentification and contamination of mouse cell lines is potentially as widespread as it is in human cell culture. This may have substantial implications for studies that are dependent on the expected background of their cell cultures. Laboratories can mitigate these risks by regular authentication of their cell cultures. Our results demonstrate that SNP array profiling is an effective method to combat cell line misidentification.

**Electronic supplementary material:**

The online version of this article (doi:10.1186/1471-2164-15-847) contains supplementary material, which is available to authorized users.

## Background

For decades, misidentified and contaminated cell lines have been a high-profile cause of wasted research effort and funding, and false claims in the literature [1–3]. In recent analyses of human and mouse cell lines, at least 13% and 4% of samples, respectively, were falsely identified [4, 5]. There is a growing movement to require the validation of all cell lines used in sponsored research [3], and some journals and repositories have heeded the call [1, 2].

Multiplex short tandem repeat (STR) profiling is the current standard for authentication of human cell lines [6], and also has been recently applied to the mouse [7]. While these assays are capable of discriminating between genetically distinct individuals and inbred strains, the long-term stability of STRs in cultured cells is in question [8]. Furthermore, STR assays may lack the resolution to discriminate between cell lines derived from closely related inbred strains [5], or to identify partial contamination, such as outbreeding that occurred prior to derivation of the cell line. Finally, STR markers cannot reliably identify chromosomal copy number aberrations that occur in culture and may have functional implications [9–11]. SNP-based assays are an attractive supplement or alternative to STR profiling that have the potential to address these limitations [8, 12–15]. Here we describe a comprehensive and cost-efficient SNP-based solution to the problems of mouse cell line misidentification, cross-contamination and copy number aberration.

## Results

### Genotype quality and reproducibility

We genotyped 117 samples from 99 commonly used mouse-derived cell lines (Additional file 1) and 503 reference samples from 245 distinct genetic backgrounds, including most commonly used inbred strains and a broad sample of outbred individuals (Additional file 2). Genotyping was done using two generations of the Mouse Universal Genotyping Array: MUGA [16] (7,800 markers) and MegaMUGA [17] (78 k markers). MegaMUGA is available commercially, and will soon transition to the third-generation GigaMUGA array (144 k markers) that is under development (JPD, FPMV, Andrew P Morgan, Leonard McMillan, Ping Fu, Katy Kao unpublished).

Considering only the 6,212 SNP markers in common between the two arrays, reference samples had a mean call rate of 94.6%. As expected, call rates varied widely (range: 52.1% – 99.5%) and were dependent on the specific and subspecific origin of the sample [18] (Additional file 3). When considering only *Mus musculus*-derived samples, call rates for reference samples (mean: 95.5%, range: 91.7 – 99.5%) were significantly higher (t-test, *p* = 0.001) and less variable (F test, *p* = 1.8×10^-5^) than for cell line samples (mean: 94.1%, range: 71.0 – 98.6%).

We measured genotype reproducibility as the fraction of markers that was fully consistent across replicates. The mean reproducibility was 0.968 (39 strains, range: 0.924 – 0.997) and 0.986 (44 strains, range: 0.970 – 0.997) for MUGA and MegaMUGA, respectively. We chose the conservative value of 0.032 for the error rate that we used in Probability of Incorrect Assignment (PIA) computations (see Methods); however, we expect that the true error rate is much lower (<0.001) when considering only the markers that pass the strict quality thresholds described below.

### Development of an assay for mouse cell line authentication

We developed an R package called Cell Line Authentication by SNP Profiling (CLASP, Additional file 4), which is described in the Methods. We used the assay development function of CLASP to select markers that met the following criteria: 1) on an autosome; 2) call rate > 80%; 3) minor allele frequency > 0 (i.e., not fixed for a single allele); 4) fully consistent across all replicate samples (regardless of genotyping platform); and 5) either not in linkage disequilibrium (LD) with adjacent markers (*r*^*2*^ < 0.25) or a different strain distribution pattern (SDP) from any linked marker. This yielded a set of 3,552 high-quality informative markers. The markers were evenly spaced across the entire autosome. Inter-SNP distances followed a Poisson distribution with median of ~500 kb (Additional file 5). Although 23% of adjacent marker pairs were in LD, the mean *r*^*2*^ value was low (0.156, Additional file 6) and no pairs had identical SDPs.

**Figure 1:**
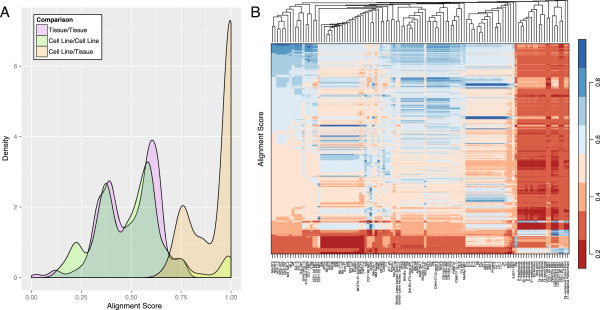
**Genotypes uniquely identify mouse strains and cell lines. A)** Density plots of alignment scores for all pairwise comparisons between reference samples (purple) and between cell lines (green), and maximum alignment scores for each cell line compared to all reference samples (orange). Alignment scores range from 0.0 (no genotypes in common) to 1.0 (genetically identical). High identity in some pairwise cell line comparisons is due to inclusion of replicates. **B)** Heat map of all comparisons between cell lines (columns) and reference samples (rows). Columns are ordered based on clustering of cell lines by genotype, as shown in the dendrogram at the top of the plot (branch lengths are arbitrary).

Among inbred strain pairs, the mean alignment score [8] (fraction of markers with identical genotypes) was 0.495 (range: 0.215 – 0.999, Figure 1 and Additional file 7). Nearly all of the 12,090 pairwise comparisons were different at 10 or more markers (corresponding to a PIA < 1.1×10^-15^). The 11 pairs with fewer than 10 differences consisted of groups of substrains (BALB/c, C57BL/6, C3H/He, DBA/1, SJL), which only differ due to genetic drift, and two wild *M. m. castaneus* mice (IN17 and IN47) that were trapped at nearby sites. We note that the ability to differentiate between closely related substrains was a key consideration in the design of the MegaMUGA array. Using the full set of MegaMUGA markers, we compared two or three different substrains from each group that our assay had trouble differentiating and found that any pair differed at a minimum of 45 markers (Additional file 8).

There were nine individuals from three outbred stocks among our reference samples, including four replicates each from the CD-1 and SW stocks. These samples were not considered during the third step of assay development (consistency check). Instead, we identified a subset of 1,652 markers that were consistent across replicates of outbred lines. The distribution of alignment scores for comparisons between the outbred stocks and the inbred/wild mice (mean alignment score: 0.564, range: 0.204 – 0.953, Additional file 9) was similar to that among only the inbred/wild mice.

### Pairwise analysis of cell lines

The mean alignment score for all pairwise comparisons among the 117 cell line samples was 0.501 (range: 0.116 – 1.0, Figure 1). Between pairs of samples with the same cell line designation, the absolute number of genotype differences was relatively high (mean: 21.3). This reflects the fact that we obtained samples from multiple repositories and/or at different passages, and suggests that there is genomic instability in some cell lines. In contrast, three replicates from the same culture (TC-1, Beverly Koller lab) were identical to each other (alignment scores of 1.0).

### Validation of strain background

**Figure 2:**
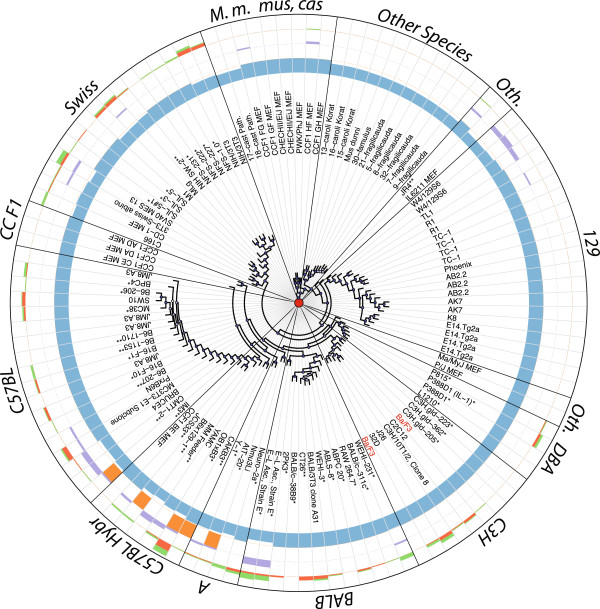
**Mouse cell lines have contamination and widespread aneuploidy.** Neighbor-joining tree of 117 cell line samples based on genotypes from 3,552 SNP markers. Node colors show the support for each clade, based on 100 resamplings (light blue = lower support, dark blue = higher support). Samples labeled in red are from the Ba/F3 cell line, which was reported to be of BALB origin but is actually derived from C3H. Asterisks denote (*) cell lines known to be derived from cancer tissue, and (**) cell lines of unknown origin. The four circular tracks (from inside to outside) show alignment score (blue), presence of a secondary genetic background (orange), cross-contamination level (purple) and number of chromosomes with evidence of copy number change (red = loss, green = gain). Labels identify groups of cell lines derived from classical inbred strains (129, A, BALB, C3H, C57BL, DBA), intercross (C57BL Hybr = hybrid between C57BL and another background, CCF1 = intercross between two Collaborative Cross (CC) founder strains), Swiss mice (including commercial outbred stocks), wild-derived strains of *M. m. musculus* or *M. m. castaneus* origin (*M. m. mus, cas*), wild mice on non-*M. musculus* origin (other species), and other backgrounds (Ma/MyJ and PL/J are classical inbred strains, IL6211 is a CC line, and JR4 is derived from a 129xCAST hybrid).

We created 6,105 *in silico* intercross samples by imputation of genotypes for all pairwise combinations of 111 *M. musculus* reference samples, which yielded 21.7 M additional genotypes. Next, we identified the best alignment score for each cell line when compared to all reference samples (primary tissue and *in silico*, mean: 0.927, range: 0.665 – 1.0, Figures 1 and 2). On average, the best match had 109 fewer genotype differences than any other reference sample (range: 1 – 1059), corresponding to a mean PIA of 1.15×10^-54^. Castro *et al.* (2012) suggest that alignment scores of 0.96 or greater are indicative of identical samples. We found that two-thirds of cell line samples matched a reference sample with an identity of at least 0.96 (Additional file 1). Of these, the best match for all but one cell line was the reported strain of origin, or a closely related strain in the event that the reported background was absent from our database or was imprecisely specified (e.g., a family of strains was reported rather than a specific substrain). The single exception, Ba/F3, most closely matched C3H/HeJ, not BALB/c as was reported. We communicated this discrepancy to RIKEN, a distributor of the Ba/F3 cell line, and they confirmed our finding [19]. They also compared the cell morphology of Ba/F3 against other C3H-derived cell lines and found that Ba/F3 was a distinct cell line rather than the result of cross-contamination.

Most cell lines with low alignment scores were derived from outbred stocks or wild-caught individuals, and thus were not expected to closely match any reference sample in our database. Each cell line derived from an outbred stock best matched an outbred stock reference sample with a mean identity of 0.86 – similar to the pairwise identity of replicate outbred reference samples. Each wild-derived cell line best matched the reference sample that was phylogenetically closest to the mouse of origin [20, 21]. We were unable to identify a single best matching reference sample for 14 cell lines (Additional file 1).

### Backcrossing and introgression

We attempted to identify the reason why our assay failed to identify a match for the 14 cell lines noted above. First, we tested whether the mismatched genotypes were due to the contribution of a second genetic background. We identified five lines of reported intercross origin (AtT-20, B6x129-1, CAKB3, IM3 and OB1xB3) that appear to have been backcrossed prior to derivation of the cell line (Figure 2). For these lines, both the best overall match and the best secondary match were to one or both of the reported intercross background with alignment scores of 0.7 or greater (OB1xB3 had a slightly lower alignment score due to known contamination by Chinese Hamster Ovary feeder cells, personal communication from Rosann Farber, Additional file 1). Additionally, these lines exhibited a non-random genomic distribution of discordant markers, which is indicative of introgression prior to the derivation of the cell line rather than contamination [22] (Additional file 10). This left nine unmatched cell lines (Additional file 1).

### Cross-contamination

**Figure 3:**
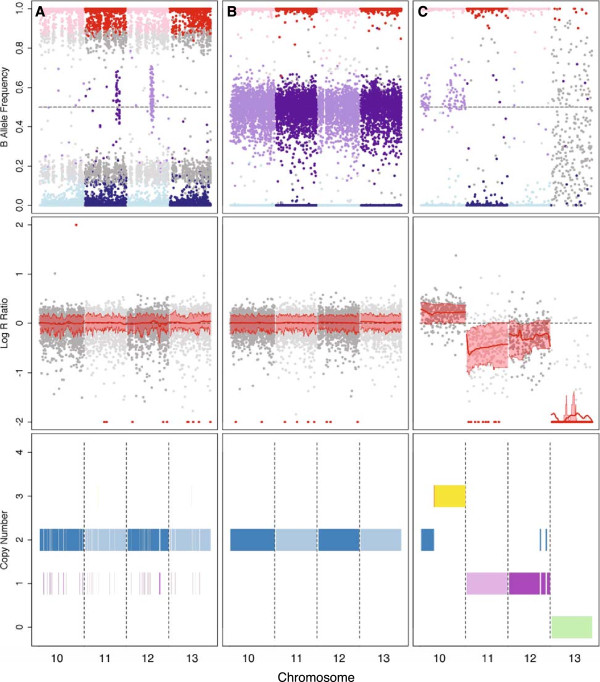
**CLASP identifies contamination and copy number aberration in cell lines.** Visualizations of genome-wide intensity distributions for **A)** a sample with cross-contamination (W4/129S6); **B)** a normal sample from primary tissue (CAST/EiJ x A/J); and **C)** an aneuploid sample (OB1xB3). Top tracks: B allele frequencies. Each data point represents a marker and is colored by genotype call, AA (blue), AB (purple) or BB (red). Middle tracks: Log R ratios. The red line is the smoothed mean LRR, and the upper and lower bands represent one standard deviation greater and lower than the mean, respectively. Markers colored red have values lying outside the range [-2,2]. Lower tracks: copy number intervals identified by genoCNA. Colors represent the different HMM states (see Sun et al. [24]).

A cell culture that is contaminated by cells of a different genetic background (Figure 3A) can be distinguished from an uncontaminated sample (Figure 3B) by visual inspection of their B allele frequencies (BAFs, the ratio of hybridization intensity values for the two allelic probes [23]). A contaminated sample exhibits a large number of markers with allelic ratios falling outside of the expected ranges. We developed a computational approach to estimate the degree of contamination, if any, in each cell line (see Methods). Our method was based on intensity distributions for each marker that we computed [23] using our reference samples. We derived the BAF thresholds for homozygous and heterozygous calls from our reference intercross samples (*T*_*hom*_ *= 0.02 and T*_*het*_ *= 0.46*).

We modeled the effect of contamination at different proportions on allelic ratios using a dilution series (ratios from 1:1 to 200:1) between Phoenix (an uncontaminated cell line of 129S6/SvEvTac origin) and a feeder cell line of unknown origin (which we treat as the contaminant). We found that the pairwise alignment scores between the pure Phoenix cells and the mixed samples decreased exponentially with the concentration of the contaminant (Additional file 11). The contaminated samples exhibited a consistent deviation from the expected BAF distribution (Additional file 12A), and there was a strong correlation between the concentration of the contaminant and the magnitude of the deviation (*r* = 0.988).

We estimated the degree of contamination in each cell line sample by fitting their mean deviations from the expected BAF distribution to a model derived from the dilution series (Additional file 12B). Of the nine unmatched samples, our method predicted that eight were cross-contaminated at ratios between 1:6 and 1:1 (Figure 2 and Additional file 1). We estimate that the minimum level of contamination that is required to observe a significant deviation from the expected BAF distribution is about 0.1 (1:10 ratio) for MUGA and 0.05 (1:20 ratio) for MegaMUGA.

### Copy number aberrations

Deviations from the expected BAF distribution may also be caused by copy number changes. For example, when one of two homologues is duplicated (trisomy), the alleles on the duplicated chromosome will be present at twice the frequency as those on the unduplicated chromosome; therefore, at heterozygous markers, a 2:1 ratio would be observed (BAF = 0.33 or 0.66). Detection of copy number variation in cell lines is complicated by two factors. First, a cell culture may be heterogeneous for a copy number variant, leading to a less intense signal than if the variant was fixed. A variant that is present only in a small fraction of cells may not produce a change in the intensity signal that is distinguishable from noise. Second, genomic regions in cells that have not undergone G1 arrest prior to DNA extraction may vary in the number of alleles depending on the cell cycle phases they are undergoing.

**Figure 4:**
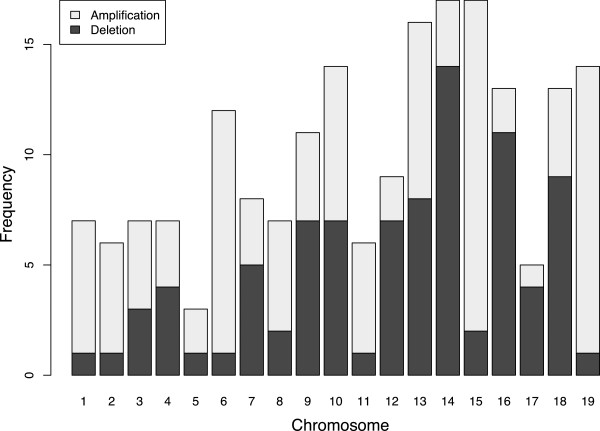
**Aneuploidy is pervasive in cell culture.** Frequency with which each chromosome was classified by genoCNA as being below our threshold for chromosome loss (mean copy number 1.5, dark gray) or above our threshold for chromosome gain (2.1, light gray).

We used the genoCNA algorithm [24] to identify copy number aberrations (CNAs) in our cell line samples (Additional file 1). We found that mean predicted copy numbers less than 1.5 and greater than 2.1 were indicative of substantial negative and positive Log R ratios (LRRs, the log transformation of the ratio between observed and expected intensities [23]), respectively, across a large portion of the chromosome. About half of cell line samples were predicted to have some degree of aneuploidy, including 24% and 62% of cell lines derived from normal and cancer tissue, respectively (Figure 2). In aggregate, there were 192 chromosomes with evidence of copy number change, roughly evenly split between loss and gain events (89 and 103, respectively, Figure 4). In most cases, however, only a fraction of the cell population appeared to be affected. Only 24 chromosomes showed evidence of complete deletion (copy number less than 1.25) or gain (copy number greater than 2.75) including three chromosomes exhibiting loss or gain of multiple copies (Additional file 1 and Figure 3C). All autosomes exhibited CNA at some level (mean: 10.1 events), although the distribution of events per chromosome was non-uniform (chi-squared test, *p* = 0.02, Figure 4).

We verified all of the predicted CNAs by visual inspection of intensity plots (Figure 3). In most cases, a mean copy number outside our specified thresholds correlated with a chromosome-wide LRR shift that was distinguishable from background noise. In a small number of cases, we identified chromosomes that were likely aneuploid, albeit at a low frequency within the cell culture, but were not identified by the algorithm (false negatives, highlighted in yellow in Additional file 1). In several instances, only a portion of the chromosome exhibited CNA (i.e., structural variation, rather than whole-chromosome loss or gain). Finally, we assessed that false-positives were rare and occurred mostly in the context of samples that exhibited cross-contamination.

Of the nine unmatched cell lines, five had multiple obvious copy number change events (mean: six events per sample, range: 2 – 10). These five lines were also those that had the least evidence for contamination. Two of these were derived from tumors (Ehrlich-Lettre Ascites Strain E and Y-1), and so the presence of aneuploidy was not surprising. The other three lines (Nmu3li, SV40 MES 13 and YAMC) were derived from primary tissue, thus the observed aneuploidy likely occurred in culture. In summary, the combination of genotype and intensity-based analysis enabled us to discriminate between multiple possible reasons for failures to verify cell line backgrounds.

## Discussion

We have compiled a database of SNP profiles for hundreds of commonly used mouse inbred strains and cell lines based on the MUGA genotyping array, which is commercially available and affordable (~US$100). The current version of the array (MegaMUGA) has an order of magnitude greater density than the initial version. Although data from both arrays are compatible, regenotyping of the samples used in this study on MegaMUGA would provide at least 1–2 orders of magnitude greater discriminatory power. We provide software (CLASP) that includes functions to manage the genotype database, perform fast and accurate identification of strain background and authentication of cell line identity, and recognize contaminants and CNAs. Although we developed this method for the purposes of cell line authentication, we expect that it will have other applications, such as the monitoring of mouse stocks used in biomedical research [25] and forensics applications (CLASP can easily accommodate human genotype data).

We found that cell lines have higher call rate variability and lower reproducibility compared to matched reference samples. This variability may be due to karyotypic rearrangements or other mutations accumulated in culture, which can alter hybridization intensities [18] and thus produce genotype calls that are different from one passage to the next. Alternatively, variability may be due to accelerated DNA replication (which creates unbalanced allelic ratios) in cell lines relative to primary tissue. It has been shown that DNA isolation procedures can greatly affect the quality of downstream data, and that inducing G1 arrest in cells prior to DNA extraction can improve results [26, 27]. Finally, converting continuous intensity data to discrete genotype calls reduces noise but also discards important information. Recent advances in methods for working directly with intensity data will enable better discriminatory power of array-based assays [28].

We found that, while most cell lines correctly matched their reported strain(s) of origin (98/99), a substantial fraction of cell lines had evidence of cross-contamination and/or aneuploidy. Our finding that 42% of cell lines tested showed evidence of aneuploidy (Figure 2 and Additional file 1) was consistent with previous findings in ES cells [10, 29]. Furthermore, aneuploidy was evident in 24% of cell lines derived from normal tissue, indicating that it is a widespread problem in cell culture and not simply a feature of cancer-derived cell lines.

We acknowledge that there are inherent limitations in applying to our data set algorithms that were designed to model human tumor data sets of 100,000 or more markers. First, the relatively low density of the MUGA platform amplified the effect of noise. The genoCNA algorithm identified many more transitions between different copy number regions in cell lines genotyped on MUGA than in the same cell lines genotyped on MegaMUGA. Second, mouse cell lines may be derived from inbred strains, which have significantly greater homozygosity than what is expected in humans. Third, no algorithm is currently capable of simultaneously modeling sample heterogeneity, cross-contamination and copy number aberration, all of which may be present in a non-clonal cell culture. While the output from genoCNA corresponded well with our visual scoring of intensity plots (Figure 3), we did not perform cytogenetic verification of any predicted CNAs. Therefore, predicted CNAs should be considered suggestive that further investigation is necessary.

## Conclusions

Misidentification and contamination of mouse cell lines is potentially as widespread as it in human cell culture. This may have substantial implications for studies that are conditioned on the expected genetic background of their cell cultures. Laboratories can mitigate these risks by regular authentication of their cell cultures. Our recommendations for future use of SNP profiling in cell line authentication are as follows. Laboratories should test their cell cultures periodically and report on changes that occur between passages. Ideally, a central database will be maintained and made accessible using the client–server capabilities of CLASP. Database maintainer(s) should obtain multiple samples of each cell line from independent labs and determine the natural variability in each cell line in order to establish cell line-specific thresholds for identity. Journals and funding agencies should require proof of authenticity for each cell line used in a study as a prerequisite for consideration. In addition, we encourage increased study of the functional impact of aneuploidy and structural variation in cell lines. While establishing the authenticity of cell lines by genetic means is an important and necessary step in establishing the validity of research findings, we hypothesize that it may not be sufficient and that verifying the structural integrity of the genome may also be necessary.

With the wide availability of STR- and SNP-based profiling, the scientific community has to the tools to resolve the long and hard-fought campaign to end the “scandalous” use of misidentified human cell lines [30]. We expect that the resources we provide here will help to extend these advances to the mouse and other model organisms.

## Methods

### Biological samples

We obtained 117 samples from 99 different cell lines in pelleted form (Additional file 1). For three cell lines (Ba/F3, E14.Tg2a and NIH/3 T3) we obtained samples from more than one source. DNA or tissue was obtained from a total of 245 distinct genetic backgrounds (Additional file 2). These included material from 89 classical inbred strains, 80 intercrosses (offspring of a cross between two inbred strains), 28 wild-derived inbred strains, 3 outbred stocks and 45 wild-caught mice.

### DNA isolation and preparation

DNA isolation was performed using the QiAmp DNA Micro Kit (Cat: 56304). Briefly, cell pellets were incubated overnight in 300 μl of lysis buffer at 65°C. The supernatant was transferred into a mixture of 300 μl of isopropanol and 0.5 μl of glycogen and DNA was isolated by centrifugation. After discarding the supernatant, the DNA pellet was washed in 70% ethanol and then resuspended in deionized water, vortexed and incubated at 55°C for one hour. The DNA concentration was determined using an ND8000 (Nanodrop) and adjusted to between 50–150 ng/μl. Samples were then randomized, and 10 μl from each sample was loaded into a 96 well plate for genotyping.

### SNP genotyping

All genotyping was done using two versions of the Mouse Universal Genotyping Array (MUGA). The initial MUGA array had 7,810 evenly spaced SNP markers. The current version of the array (MegaMUGA) has 77,808 SNP markers. Array processing and genotype calling were performed by GeneSeek/Neogen (http://neogen.com) as previously described [16].

Genotype QC was performed by clustering H and N call rates by sample type and species/subspecies and removing outliers. For reference samples, outliers were defined as being outside 1.5 times the interquartile range. For cell lines, we could not estimate the expected H and N rates; therefore, we eliminated only extreme outlier samples by visual examination. Second, we calculated the Kolmogorov-Smirnov statistic (*D*) of the sum intensity distribution for each sample compared to a reference distribution estimated from a larger set of reference samples. The sum intensity for each bi-allelic probe is *I = X + Y*, where *X* and *Y* are the normalized intensity values for the two alleles. Outliers for *D* fell into two categories: those with left-shifted distributions and those with normal distributions but a “spike” at *I ~ 0*. The former are recognized as genotyping failures and were eliminated, while the later were associated with species other than *M. musculus*. The “spike” in the later distributions was expected, as it represented probe sequences that were not sufficiently conserved in the other species and thus resulted in the absence of hybridization signal.

After eliminating poor-quality arrays, our database contained genotypes for 620 samples. For ease of data integration and interpretation, only the 6,212 autosomal SNPs common to both arrays were used for analysis; however, the complete set of genotypes is available in the database.

### Dilution series

We isolated DNA from pellets of the Phoenix cell line (Anne Latour and Beverly Koller unpublished), and of a mouse embryo fibroblast “feeder” cell line of unspecified *M. musculus* background. We normalized the concentrations of both samples to ~15 ng/μl using a Qubit Fluorometer (Invitrogen). We then created seven mixtures of Phoenix/Feeder with final volumes of 100 μl each, as follows: 100/0, 90/10, 75/25, 50/50, 25/75, 10/90, 0/100. These mixtures were genotyped as described above.

### CLASP

A complete description of the CLASP framework is available in the documentation for the R software package. Briefly, CLASP provides three main functions that are agnostic of the details of the origin or coding of the genotype data (Additional file 4). 1) Genotype data are recoded and imported along with sample and SNP annotations into an SQLite relational database (http://www.sqlite.org). Optionally, the database can be expanded to include *in silico* intercrosses created by imputation from pairwise combinations of reference sample genotypes. 2) Genotype data are analyzed to identify the subset of SNP markers that are most reliable and informative for authentication purposes. Reliability is determined by the consistency of genotypes across replicate samples, while informativeness is determined by allele frequencies. This step is controlled by many parameters, including criteria for filtering based on Hardy-Weinberg equilibrium (HWE) and linkage disequilibrium (LD). 3) All reference and cell line sample data are analyzed simultaneously to identify the best match for each experimental sample. Additionally, when hybridization intensity data is available, CLASP can utilize the genoCNA algorithm [24] to identify allelic imbalances and copy number changes in cell lines.

Samples from outbred mice require special consideration because, although each outbred individual is genetically unique, individuals are not uniquely identified. CLASP does not exclude markers that are inconsistent across samples of the same outbred line, and instead maintains a separate list of the subset of markers that are consistent within outbred lines. This smaller list of markers is only used when comparing an outbred line against another sample.

Authenticated experimental samples may be added to the database to serve as references in future applications of the assay. Multiple databases can be merged, enabling labs to share results easily. Alternately, the software can be configured to run in a client–server environment to enable a central authority to maintain a canonical database.

### Statistics

For forensic applications, Random Match Probability (RMP) [31] is the standard measure of an assay’s discriminatory power. However, RMP is an insufficient metric when the assay must discriminate between distinct yet highly related genetic backgrounds (e.g. sister lines of inbred mouse strains). Consider, for example, a set of 1000 unlinked markers. The RMP for this marker set is 3×10^-1000^ (essentially zero). However, two sister lines that differ at only 0.1% of markers will have haplotypes that differ at only a single marker. The ability to discriminate between these two lines depends entirely on the probability that the observed haplotypes reflect the true haplotypes with complete accuracy.

We propose a new metric, the Probability of Incorrect Assignment (PIA). PIA depends on two variables: the error rate of the genotype data (*E*) and a pairwise haplotype difference matrix (*H*) for all samples being compared. The PIA for a pair of samples (*i, j*) is the probability that all of the genotypes that differentiate two samples have been incorrectly ascertained. CLASP assigns each match a PIA that is simply the maximum PIA for that sample compared to all the other samples in *H*:


For each match result, CLASP also returns an alignment score, which is simply the percent haplotype identity between the experimental sample and the closest matching reference sample. When the alignment score falls below a specified threshold, CLASP attempts to identify the contribution of a second genetic background (due to hybridization, introgression or contamination) using only the genotypes that are inconsistent with the closest matching reference sample.

### Intensity normalization

Hybridization intensity data is subject to multiple classes of noise that can be attenuated by normalization procedures. We employed two normalization steps for BAFs and LRRs (these values can be computed automatically by Illumina BeadStudio software, but they were not available in our data files). First, we used thresholded quantile normalization (tQN) to correct for dye bias, which is specific to the Illumina platform [32]. Second, we adjusted for “genomic waves” – variations in intensity that are caused by differences in local DNA quantity and are indicated by GC content [33] – using the genomic_wave.pl script of PennCNV [34].

### Allelic imbalance

BAFs are in the range [0,1] and are normally distributed (with standard deviation determined by the sample noise) around 0, 0.5 and 1 when alleles are present in the expected ratios of 2:0, 1:1 or 0:2 for AA, AB and BB genotypes, respectively. When a contaminant introduces additional alleles and has a different genotype than the host sample, it changes the allelic ratio. If the degree of contamination is high enough, the deviation can be distinguished from background noise.

Given thresholds *T*_*hom*_ and *T*_*het*_ representing the normal range of homozygous and heterozygous BAF values, we first transform the BAF value for each marker. Next, we compute the deviation of each sample from the expected BAF distribution as the sum of the transformed, non-zero BAFs divided by the total number of markers (*M)*:


### Copy number aberrations

We use the genoCNA function of the genoCN R package [24] to identify CNAs (amplification or deletion of chromosomal regions). This function requires two platform-specific parameters, distThreshold (the maximum distance, in bp, between adjacent SNPs) and geno.error (the estimated genotyping error rate). We set distThreshold at 750,000 and 100,000 and geno.error at 0.03 and 0.01 for MUGA and MegaMUGA, respectively. The output of this algorithm is a list of genomic intervals for which copy number could be inferred. In intervals where genoCNA could not predict the copy number, we assumed a copy number of 2.

### Availability of supporting data

The CLASP R package is available in CRAN (http://cran.r-project.org). Supporting data has been deposited in the figshare repository (http://dx.doi.org/10.6084/m9.figshare.1185417).

## Electronic supplementary material

Additional file 1: **Analysis of 117 cell line samples.** Two worksheets show 1) results of analysis by 611 CLASP and 2) column annotations for the first worksheet (XLSX 116 KB)

Additional file 2: **Analysis of 503 reference samples.** Four worksheets list reference samples obtained from 1) inbred strains, 2) outbred stocks, 3) F1 hybrids between two inbred strains and 4) wild-caught mice. A fifth worksheet summarizes the level of consistency between biological/technical replicates. (XLSX 89 KB)

Additional file 3: **Call rates depend on type and taxon of samples.** Heterozygous (H) call rate (x-axis) and No-call (N) rate (y-axis) out of 6,212 markers for 620 samples. Color represents sample type: primary (blue) or cell line (orange). Shape represents sample taxonomy: *M. musculus* (circle) or other (square). Inset: primary samples have higher variability in H call rate, but generally lower N call rates (excluding non-*M. musculus* samples). (PDF 3 MB)

Additional file 4: **Overview of CLASP software.** Closed rectangle: processes; cylinder: database; open rectangle: input data sets; trapezoid: output data set. (PDF 46 KB)

Additional file 5: **MUGA has sub-chromosome resolution to detect contamination and copy number variation.** Density plot of distances between all adjacent pairs of 3,552 informative markers. Most inter-SNP distances are < 2 Mb, meaning MUGA can detect contaminations and copy number variants on the order of tens-of-megabases. (PDF 240 KB)

Additional file 6: **Most MUGA marker pairs are unlinked.** Histogram of *r* ^*2*^ values for all adjacent pairs of 3,552 informative markers. Typical thresholds used to classify linkage disequilibrium are 0.3 – 0.7. (PDF 185 KB)

Additional file 7: **Most reference samples are uniquely identified by SNP profile.** Heatmap of pairwise comparisons between reference samples. Each point represents the number of genotype differences (out of 3,552) between the pair. (PDF 140 KB)

Additional file 8: **Pairwise comparison of sister strain differences in MegaMUGA genotypes.** (XLSX 38 KB)

Additional file 9: **Outbred stocks are genetically distinct from inbred strains.** Histogram of pairwise alignment scores between nine outbred individuals and 156 inbred strains. (PDF 5 KB)

Additional file 10: **Cell lines from introgressed backgrounds have non-random differences from best matching reference sample.** Physical locations of markers for which cell line genotypes don’t match the reported strain of origin for A) CAKB3, a cell line derived from an animal of a mixed (i.e., non-pure inbred strain) genetic background, and B) W4129, an apparently contaminated cell line. (PDF 2 MB)

Additional file 11: **Alignment score is negatively correlated with level of contamination.** Top panel shows relative concentrations of Phoenix cell line (blue) and a contaminating feeder line (yellow) in a dilution series experiment. Bottom panel shows H (gray) and N (green) call rates (left y-axis) and alignment scores (red line, right y-axis) between the mixtures and the pure Phoenix cell line sample (left-most sample, i.e. “Phoenix_100_Feeder_0”). (PDF 326 KB)

Additional file 12: **BAF deviation accurately predicts fraction of contaminant.** A) In the dilution series, there is a direct relationship between the fraction of contaminant (i.e. the feeder line) and the shift in BAF from the expected distribution. B) A model derived from the dilution series (red circles) enables prediction of the fraction of contamination in cell lines genotyped on MUGA (blue diamonds) and MegaMUGA (green squares) based on BAF deviation. (PDF 300 KB)

## Abbreviations

BAF: B Allele frequency
CNA: Copy number aberration
LD: Linkage disequilibrium
LRR: Log R Ratio
PIA: Probability of incorrect assignment
SDP: Strain distribution pattern
SNP: Single nucleotide polymorphism
STR: Short tandem repeat.
