# Supplementary figures and images for: SNP array profiling of mouse cell lines identifies their strains of origin and reveals cross-contamination and widespread aneuploidy
Source: BMC Genomics. 2014 Oct 3;15(1):847. doi: 10.1186/1471-2164-15-847 (PMC4198738; doi:10.1186/1471-2164-15-847)

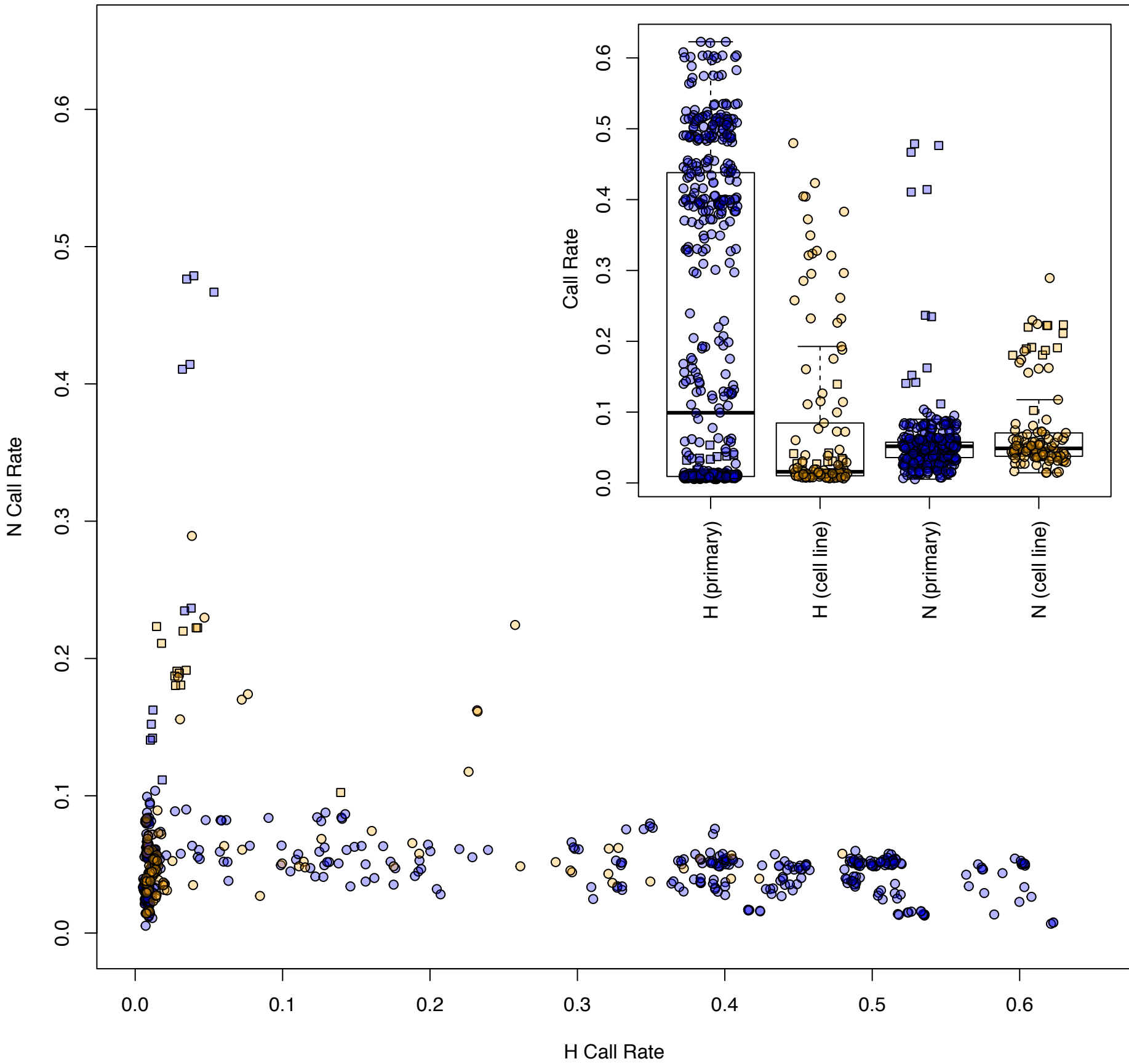

Supplement: Supplementary file 3 — Additional file 3: Call rates depend on type and taxon of samples. Heterozygous (H) call rate (x-axis) and No-call (N) rate (y-axis) out of 6,212 markers for 620 samples. Color represents sample type: primary (blue) or cell line (orange). Shape represents sample taxonomy: M. musculus (circle) or other (square). Inset: primary samples have higher variability in H call rate, but generally lower N call rates (excluding non-M. musculus samples). (PDF 3 MB) [file 12864_2014_6544_MOESM3_ESM.pdf]

1)

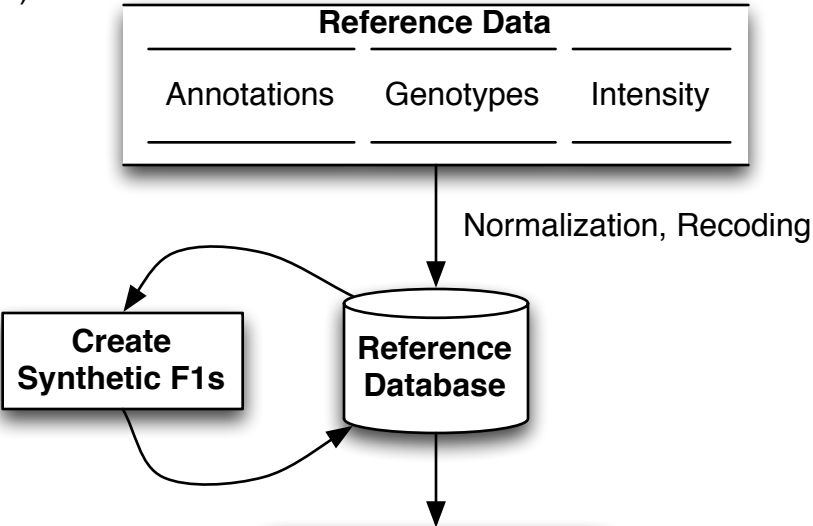

2)

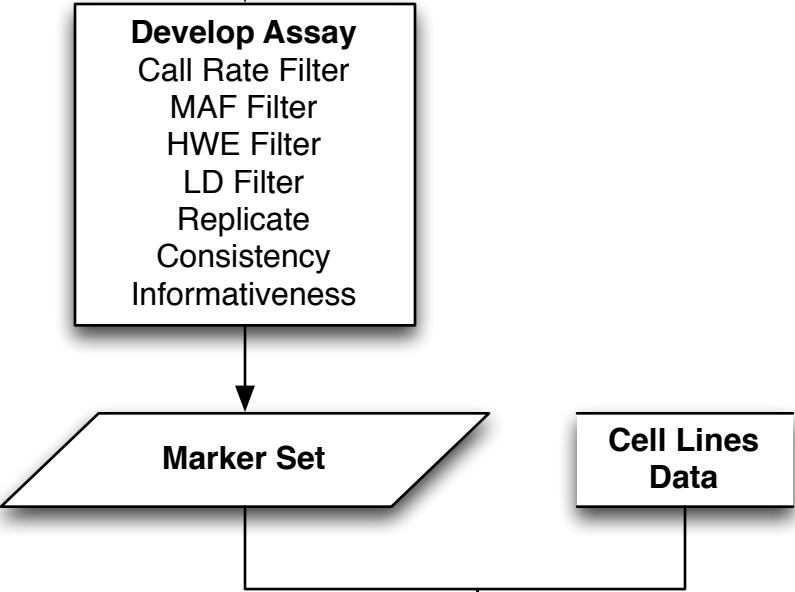

3)

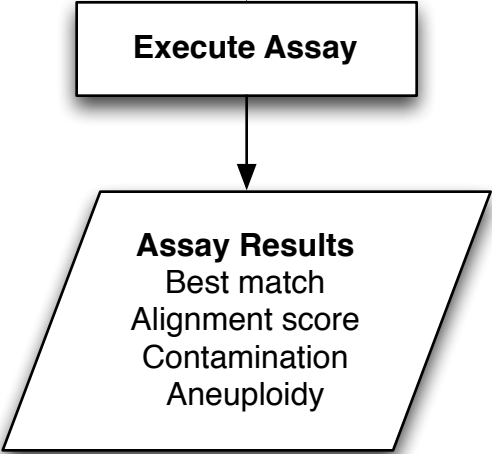

Supplement: Supplementary file 4 — Additional file 4: Overview of CLASP software. Closed rectangle: processes; cylinder: database; open rectangle: input data sets; trapezoid: output data set. (PDF 46 KB) [file 12864_2014_6544_MOESM4_ESM.pdf]

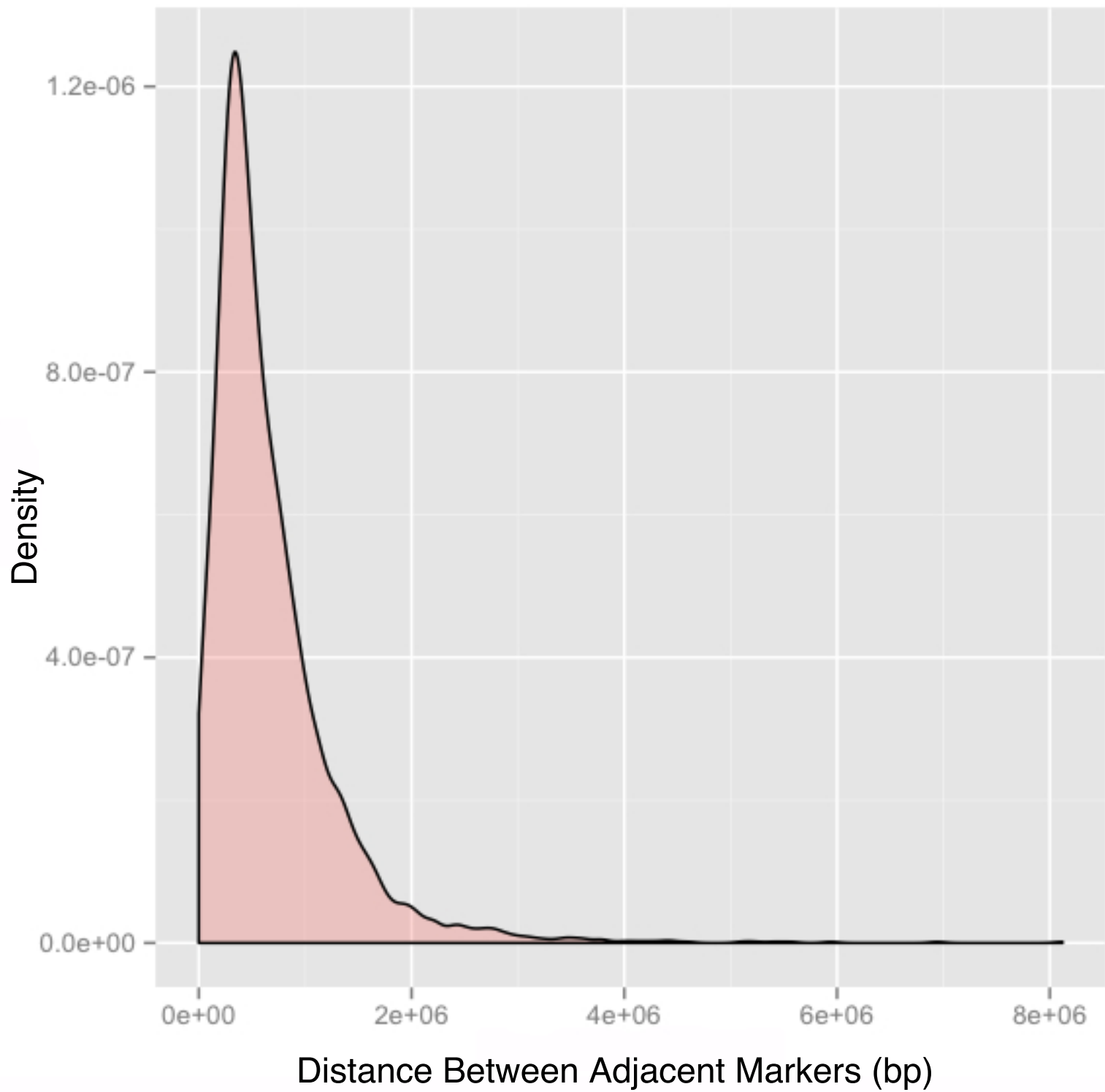

Supplement: Supplementary file 5 — Additional file 5: MUGA has sub-chromosome resolution to detect contamination and copy number variation. Density plot of distances between all adjacent pairs of 3,552 informative markers. Most inter-SNP distances are < 2 Mb, meaning MUGA can detect contaminations and copy number variants on the order of tens-of-megabases. (PDF 240 KB) [file 12864_2014_6544_MOESM5_ESM.pdf]

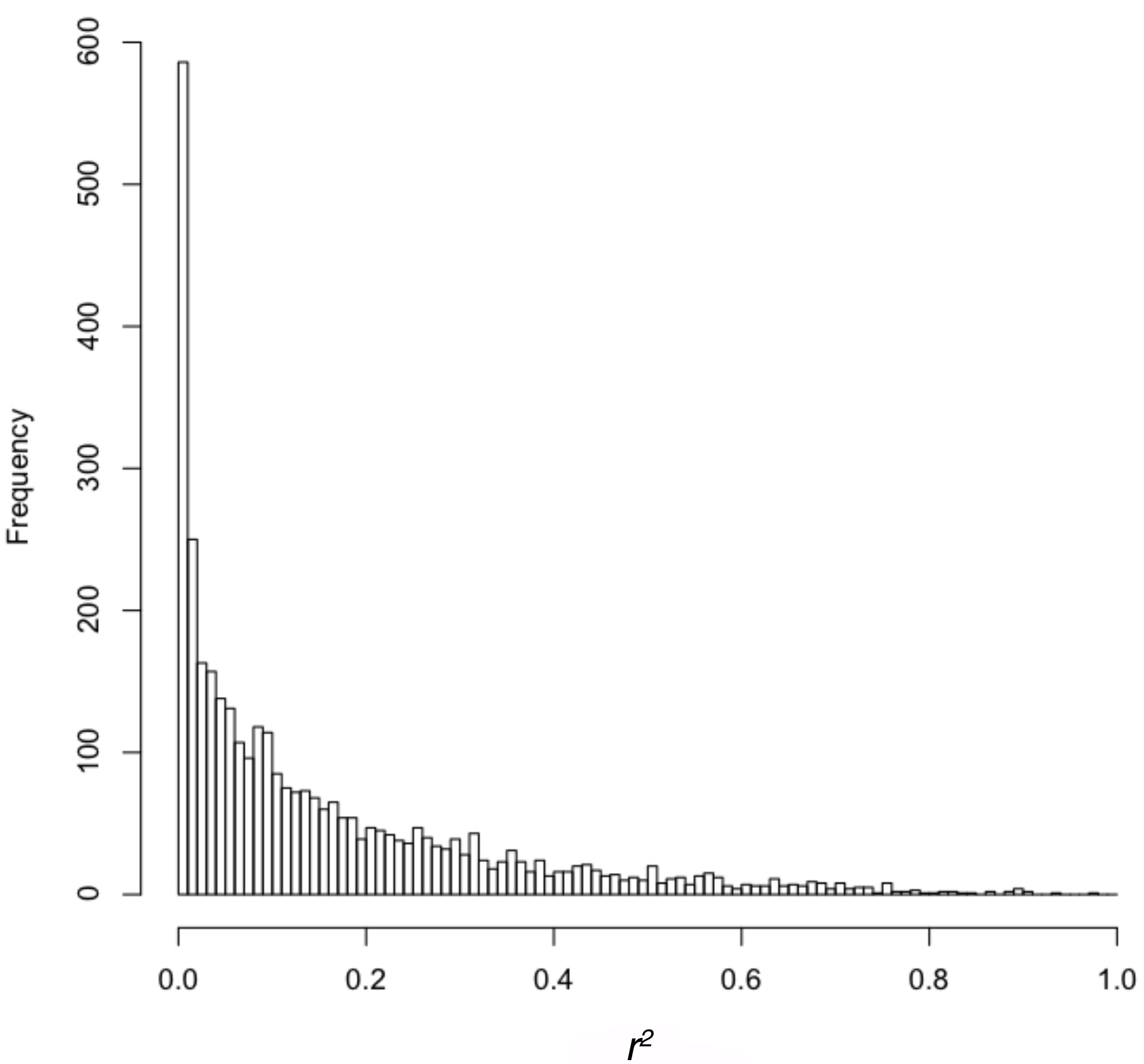

Supplement: Supplementary file 6 — Additional file 6: Most MUGA marker pairs are unlinked. Histogram of r 2 values for all adjacent pairs of 3,552 informative markers. Typical thresholds used to classify linkage disequilibrium are 0.3 – 0.7. (PDF 185 KB) [file 12864_2014_6544_MOESM6_ESM.pdf]

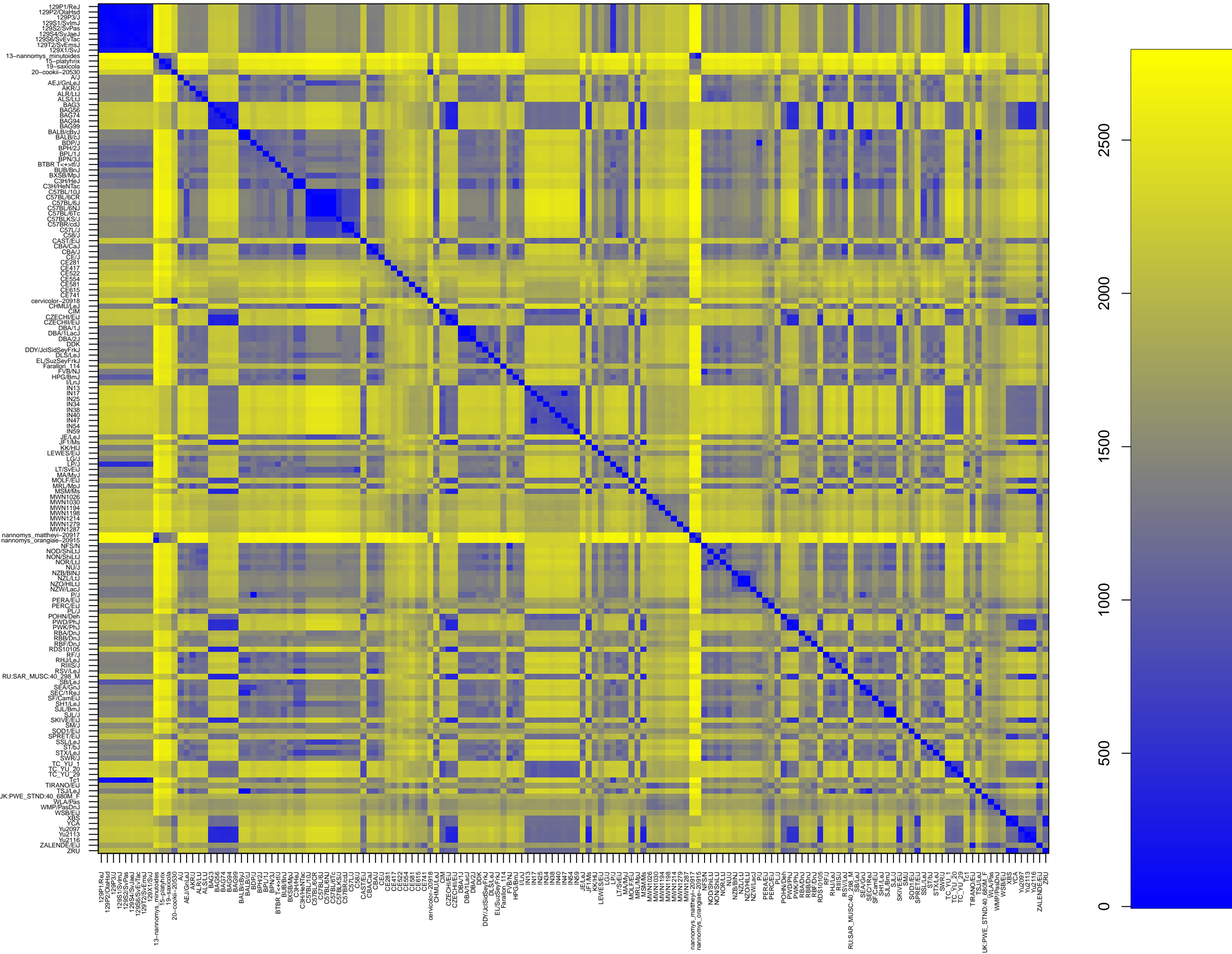

Supplement: Supplementary file 7 — Additional file 7: Most reference samples are uniquely identified by SNP profile. Heatmap of pairwise comparisons between reference samples. Each point represents the number of genotype differences (out of 3,552) between the pair. (PDF 140 KB) [file 12864_2014_6544_MOESM7_ESM.pdf]

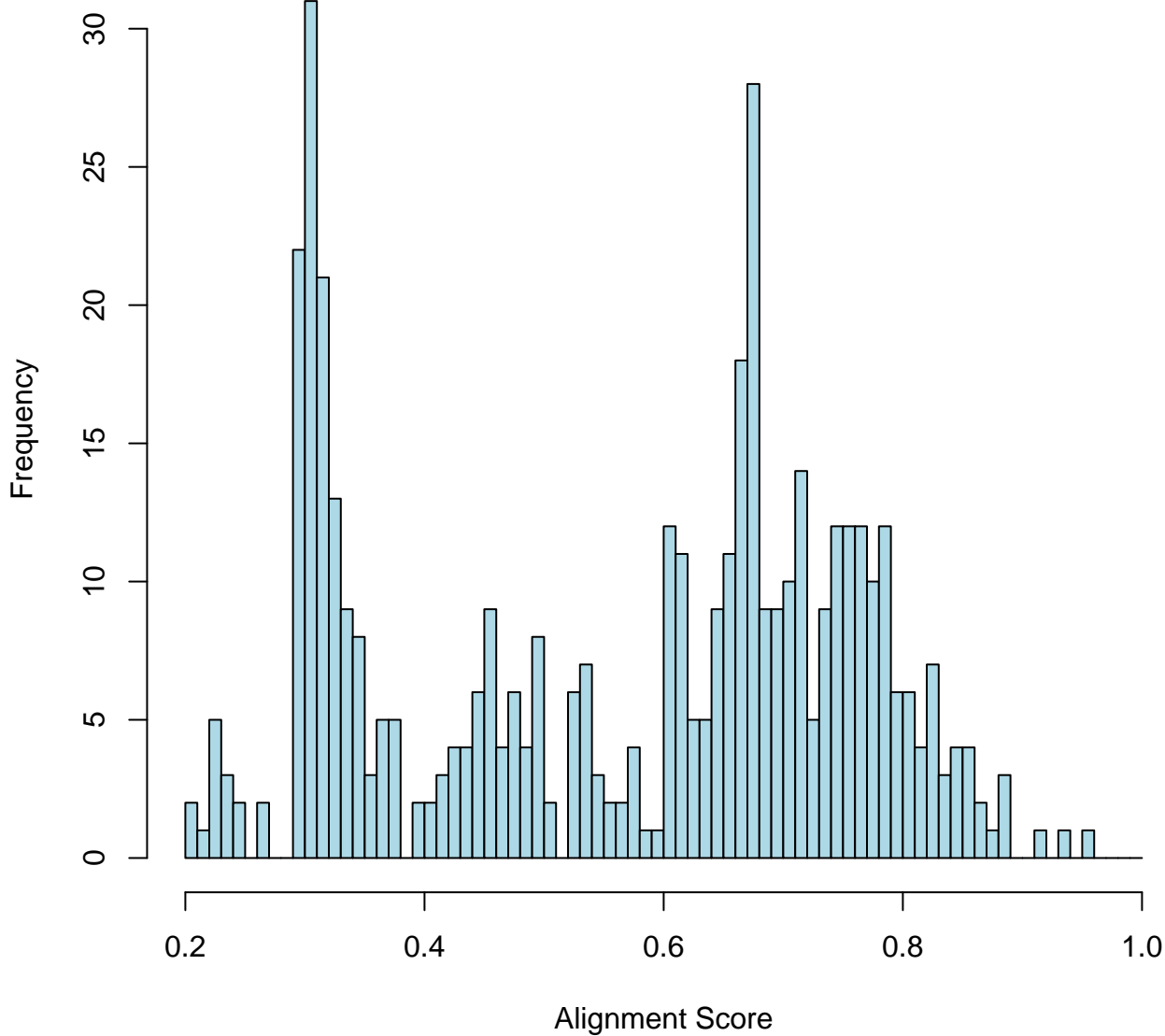

Supplement: Supplementary file 9 — Additional file 9: Outbred stocks are genetically distinct from inbred strains. Histogram of pairwise alignment scores between nine outbred individuals and 156 inbred strains. (PDF 5 KB) [file 12864_2014_6544_MOESM9_ESM.pdf]

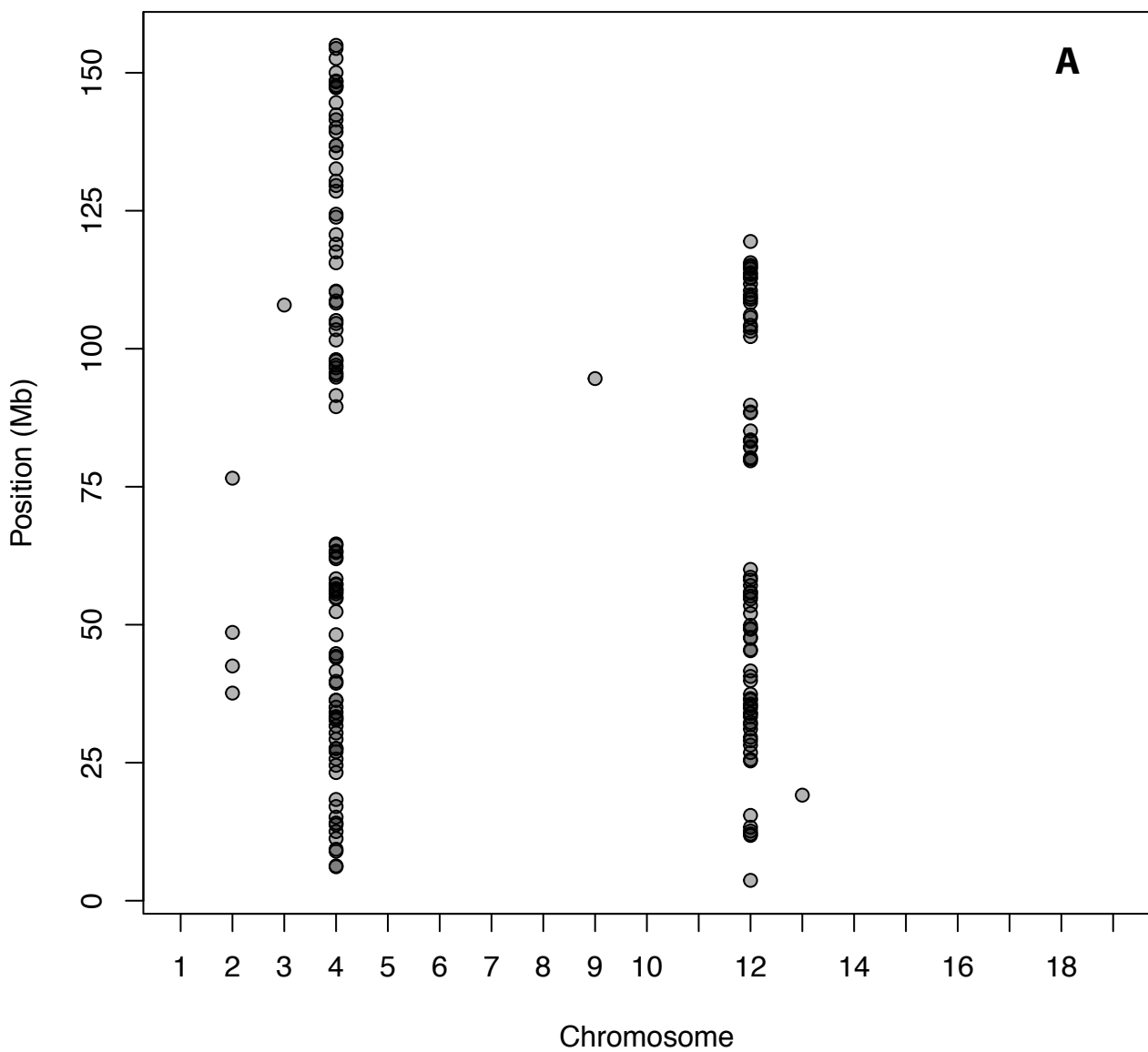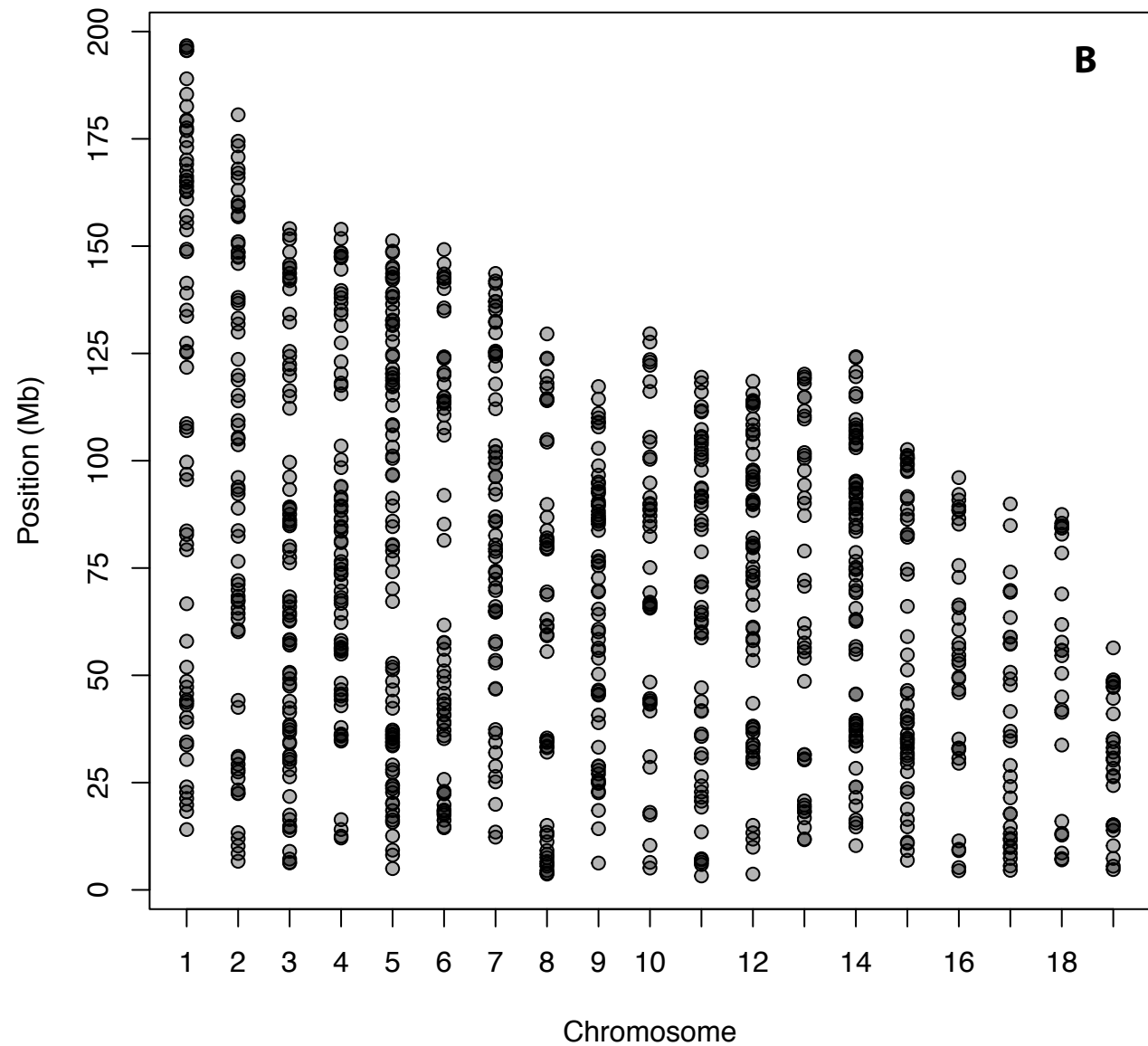

Supplement: Supplementary file 10 — Additional file 10: Cell lines from introgressed backgrounds have non-random differences from best matching reference sample. Physical locations of markers for which cell line genotypes don’t match the reported strain of origin for A) CAKB3, a cell line derived from an animal of a mixed (i.e., non-pure inbred strain) genetic background, and B) W4129, an apparently contaminated cell line. (PDF 2 MB) [file 12864_2014_6544_MOESM10_ESM.pdf]

Call Rate

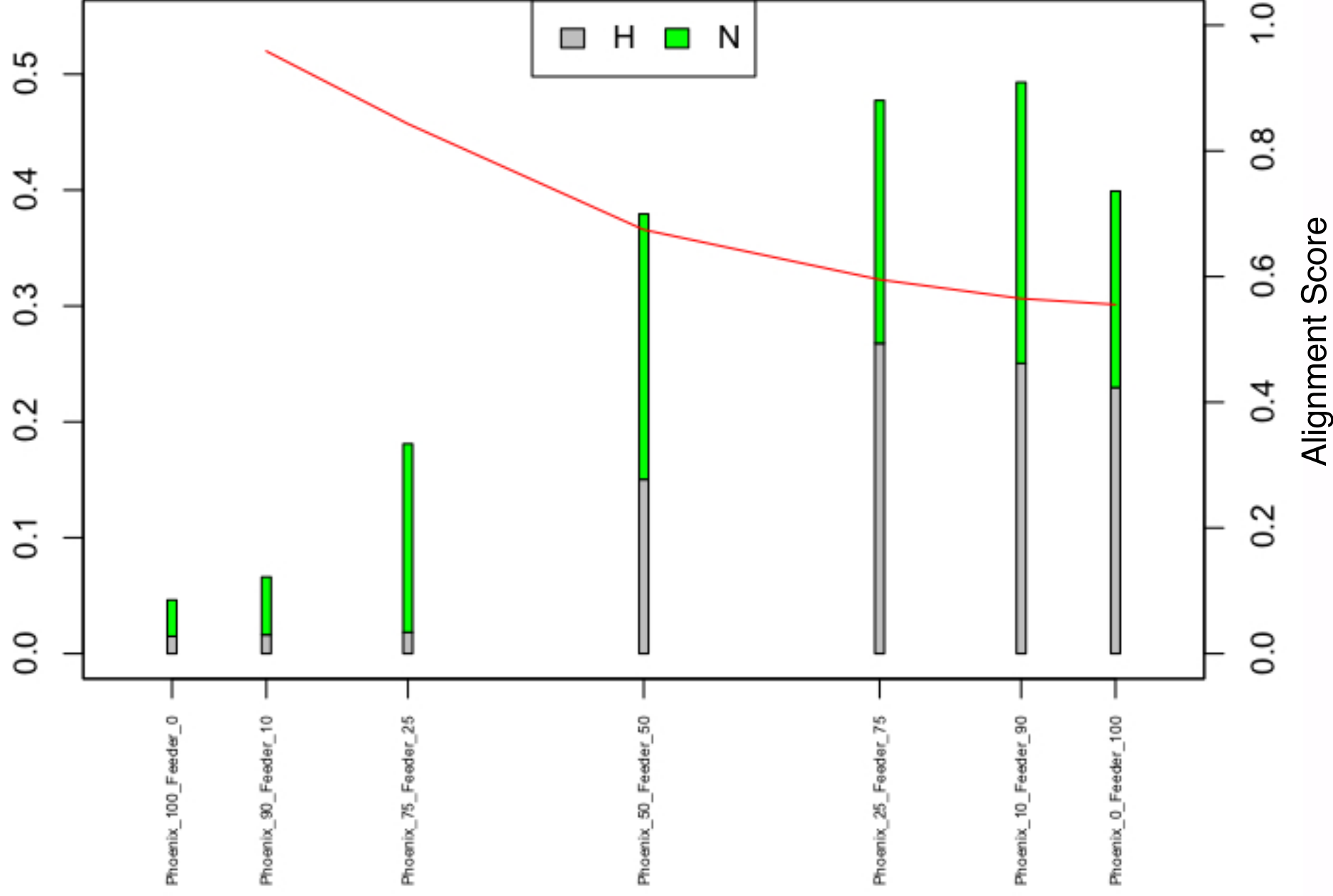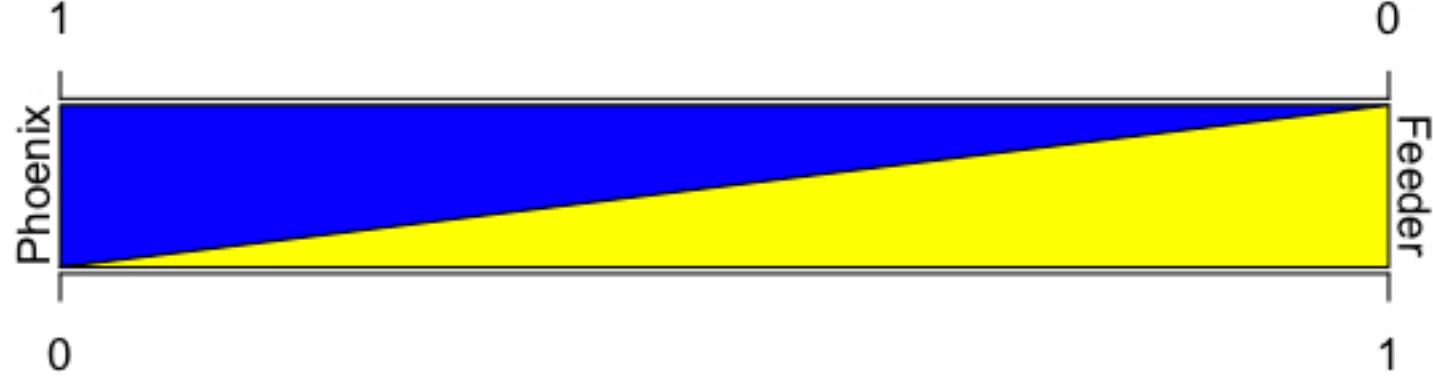

Supplement: Supplementary file 11 — Additional file 11: Alignment score is negatively correlated with level of contamination. Top panel shows relative concentrations of Phoenix cell line (blue) and a contaminating feeder line (yellow) in a dilution series experiment. Bottom panel shows H (gray) and N (green) call rates (left y-axis) and alignment scores (red line, right y-axis) between the mixtures and the pure Phoenix cell line sample (left-most sample, i.e. “Phoenix_100_Feeder_0”). (PDF 326 KB) [file 12864_2014_6544_MOESM11_ESM.pdf]

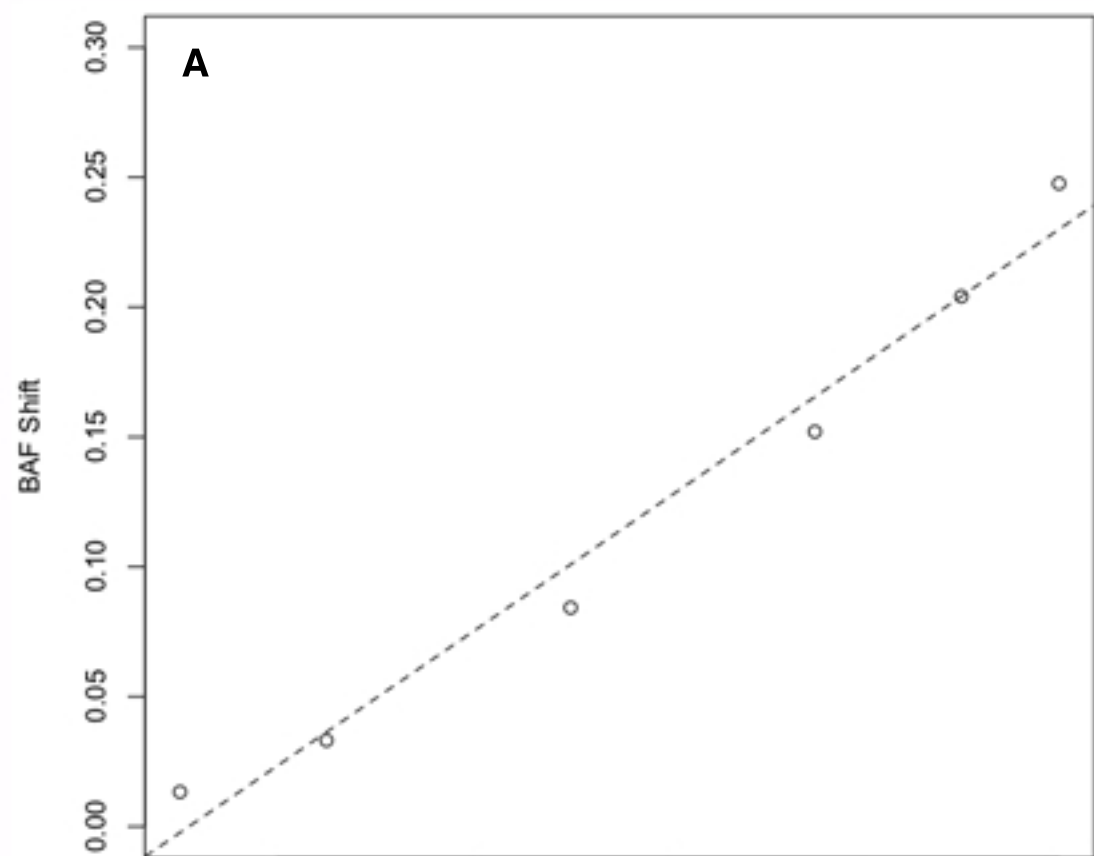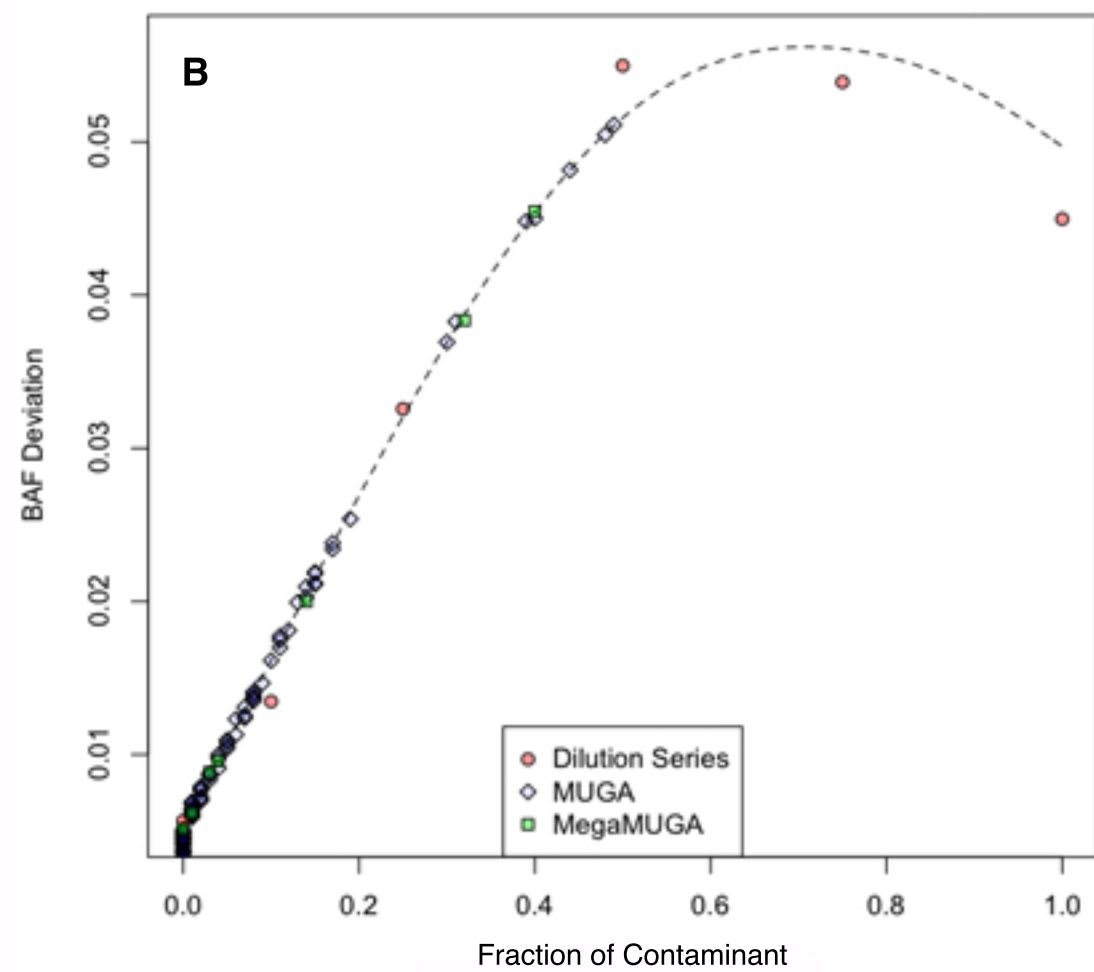

Supplement: Supplementary file 12 — Additional file 12: BAF deviation accurately predicts fraction of contaminant. A) In the dilution series, there is a direct relationship between the fraction of contaminant (i.e. the feeder line) and the shift in BAF from the expected distribution. B) A model derived from the dilution series (red circles) enables prediction of the fraction of contamination in cell lines genotyped on MUGA (blue diamonds) and MegaMUGA (green squares) based on BAF deviation. (PDF 300 KB) [file 12864_2014_6544_MOESM12_ESM.pdf]
